# Defect detection method of printed circuit boards based on EDF-YOLOv10

**DOI:** 10.1371/journal.pone.0343130

**Published:** 2026-03-06

**Authors:** Zhijuan Shen, Yonger Yao, Lin Liu, Yiqing Cao, Lijun Lu

**Affiliations:** 1 School of Mechanical, Electronic & Information Engineering, Putian University, Putian, Fujian, China; 2 School of Mechatronic Engineering and Automation, Shanghai University, Shanghai, China; King Fahd University of Petroleum & Minerals, SAUDI ARABIA

## Abstract

To address the challenges of inadequate feature representation for small objects and slow model convergence in printed circuit board (PCB) defect detection, this paper proposes an improved YOLOv10 algorithm and develops a real-time detection system with a co-optimized hardware and software architecture. The efficient channel attention (ECA) mechanism is used to enhance the ability of the model to extract key channel features; the dynamic snake convolution (DSConv) in the backbone strengthens the model’s capacity to recognize the geometric structures of small targets through deformable kernels and multi-directional feature fusion; the Focaler-CIoU loss emphasizes samples with low intersection over union (IoU) values to boost hard sample learning and improve convergence efficiency. To simulate real-world industrial environments, multiple data augmentation strategies are utilized to expand the PKU-Market-PCB dataset, thereby enhancing the model’s generalization and robustness in complex scenarios. Experimental results demonstrate that the proposed EDF-YOLOv10 achieves mAP@0.50 of 90.6% and mAP@0.50:0.95 of 48.4% on the experimental dataset, representing improvements of 3.0 and 1.6 percentage points over the baseline, respectively. Furthermore, We also develope a real-time interactive detection system for identifying PCB defects. This system utilizes industrial cameras, a controllable light source, and a graphical user interface developed with the PyQt5 framework, employing the EDF-YOLOv10 model. Our approach serves as a methodological reference for detecting PCB defects in complex industrial environments.

## 1. Introduction

Printed circuit boards (PCBs) are fundamental components in electronic systems and are widely used in communication devices, medical equipment, aerospace technology, industrial control systems, and various other applications. During the manufacturing process, defects such as open circuits, shorts, and missing holes can occur on the surface of the PCB. These defects can lead to operational failures in electronic devices, ultimately affecting their service life. As the world increasingly embraces intelligent technology, electronic products are evolving towards higher performance, miniaturization, and multi-functionality [1–3]. As a result, there is a growing demand for high-precision PCBs. Therefore, efficient detection of surface defects is essential to ensure PCB quality and enhance production efficiency.

Traditional techniques for detecting surface defects in PCBs mainly include manual visual inspection, electrical testing, and infrared testing [4,5]. Manual visual inspection requires operators to check products on the production line one by one; this method is inefficient and prone to both missed detections and false positives [6–8]. Electrical testing employs a contact-based detection principle, establishing a physical connection with the PCB through a bed of nails or probes. However, the pressure from the contacts can cause damage to the pads. Infrared testing has limited effectiveness in detecting defects unrelated to thermal issues, and environmental factors can impact its accuracy. In the industrial field, advances in intelligent detection, signal processing and optimization technologies have become key support for breaking through the limitations of traditional detection methods [9–11].With advancements in computer and machine learning technologies, machine vision-based PCB detection has emerged as an important inspection method for many manufacturing enterprises due to its low cost and ease of use [12,13]. This technology captures image data of the PCB surface using optical systems and then applies machine learning algorithms for defect classification and localization. Traditional machine learning algorithms involve manually extracting surface features and training classifiers to recognize defects. However, these methods are often affected by human subjectivity, have poor generalization capabilities, and struggle to adapt to complex industrial scenarios.

Deep learning algorithms surpass traditional machine learning techniques in autonomously identifying features of surface defects, reducing human subjectivity and improving detection accuracy. Hu et al. improved the Faster R-CNN network by integrating ResNet50 with a feature pyramid network (FPN) to extract features of small targets on PCBs [14]. Their approach utilized a GARPN for anchor prediction and incorporated residual units from ShuffleNetV2. Kim et al. proposed a PCB inspection system based on a skip-connected convolutional auto-encoder. This model decodes non-defect images from defective ones and localizes defects by comparing them with original images, achieving a detection rate of 98.0% on experimental datasets [15]. Lim et al. improved YOLOv5’s FPN and optimized the CIoU (Complete Intersection over Union) loss function, enhancing detection accuracy for tiny and multi-scale defects on PCB surfaces [16]. Xiao et al. introduced a coordinate attention mechanism (CA), DSConv, and Inner-CIoU loss function to refine the YOLOv7-tiny algorithm, attaining a mean average precision (mAP) of 98.3% on PCB datasets [17]. Wu et al. developed a defect detection method based on an improved YOLOv4, and they optimized anchor determination strategies and incorporated MobileNetV3 and Inceptionv3 networks to elevate PCB surface inspection accuracy [18]. Mao et al. enhanced the YOLOv8 algorithm by integrating a Swin Transformer module, a global attention mechanism, and a WIoU loss function, thereby increasing detection accuracy by 2.42 percentage points on the DeepPCB dataset [19].

Despite the advancement of improved deep learning methods in PCB defect detection technology, existing research still struggles to solve the core problems in PCB defect detection. General attention modules and convolutional structures often fail to capture subtle defect features or conform to irregular contours effectively, resulting in high miss rates for small defects and poor localization of irregular ones. Moreover, existing improvements often increase model complexity through module redundancy, failing to meet the dual industrial requirements for lightweight design and high accuracy. This study introduces the efficient channel Attention (ECA) mechanism, the dynamic snake convolution (DSConv) module, and the Focaler-CIoU loss function to enhance the detection accuracy of PCB surface defects using the YOLOv10 model. Furthermore, we design a PCB defect detection system based on the improved EDF-YOLOv10 algorithm.

The key contributions of this study are outlined as follows: This paper introduces the ECA mechanism, allowing the model to prioritize important channel features while reducing the influence of less significant ones; integrates the DSCC2f module into the backbone network to improve the model’s ability to extract features from irregularly shaped defects by incorporating the DSConv module; develops the Focaler-CIoU loss function by integrating the Focaler-IoU with the CIoU method, adaptively increasing the weight of low-IoU samples during training. This modification improves the detection performance for hard samples, and finally proposes EDF-YOLOv10 algorithm, combined with the developed hardware system, establishes a real-time PCB inspection system for defect detection.

## 2. Dataset

**Fig 1:**
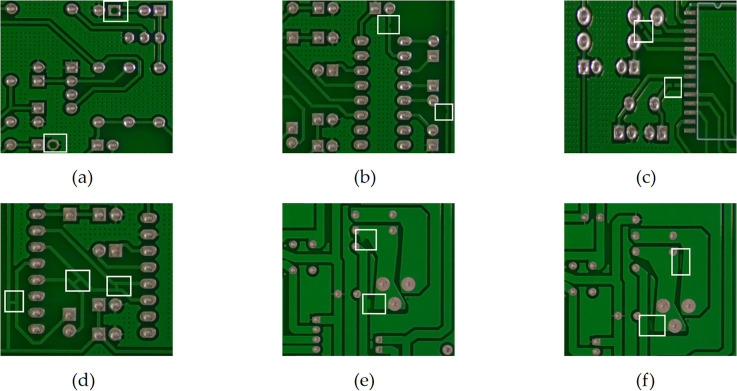
PCB dataset defect types. **(a)** missing hole; **(b)** mouse bite; **(c)** open circuit; **(d)** short; **(e)** spur; **(f)** spurious copper.

**Fig 2:**
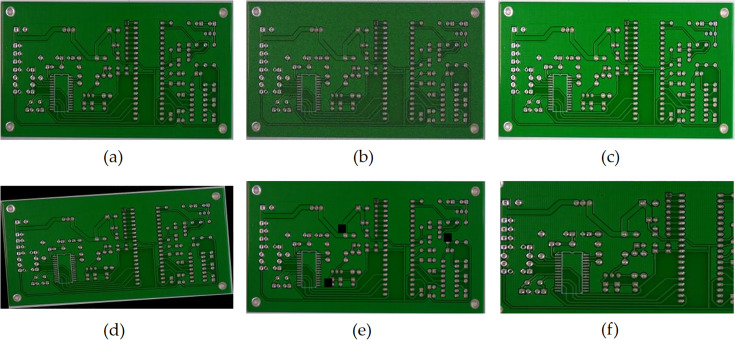
PCB dataset augmentation methods. **(a)** original figure; **(b)** noise injection; **(c)** brightness variation; **(d)** rotating; **(e)** cutout, **(f)** cropping.

This study uses the publicly available PKU-Market-PCB dataset from Peking University [20]. The dataset consists of a total of 1386 images, which encompass six types of PCB defects: missing hole, mouse bite, open circuit, short, spur, and spurious copper. The characteristics of these defects are illustrated in Fig 1. To enhance the model’s generalization ability in complex industrial environments, we applied data augmentation techniques to the training set. These techniques included noise injection, brightness variation, rotation, cutout, and cropping, with examples of the augmented images presented in Fig 2. The experimental dataset is divided into a training set containing 2,208 images, a validation set with 78 images, and a test set comprising 63 images.

## 3. YOLOv10 algorithm

**Fig 3:**
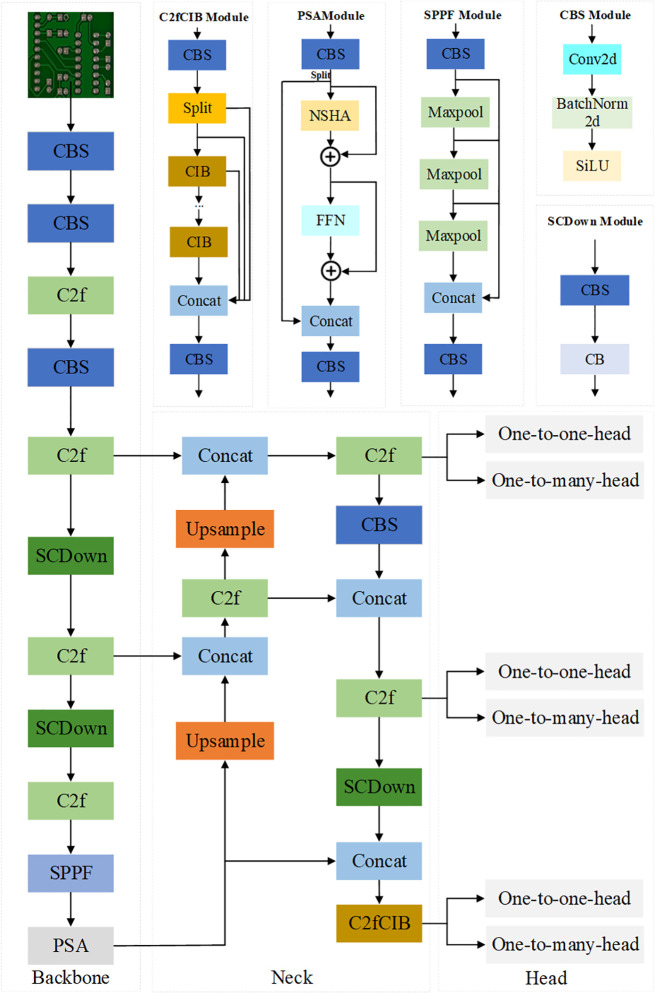
Structure of YOLOv10n network.

The YOLOv10 algorithm includes five model variants: YOLOv10n, YOLOv10s, YOLOv10m, YOLOv10l, and YOLOv10x. These variants are designed for object detection tasks in different application scenarios [21]. For PCB defect detection, which requires high efficiency and a lightweight design, this paper selects YOLOv10n as the baseline model due to its minimal number of parameters. The network structure of YOLOv10n, illustrated in Fig 3, consists of three main components: Backbone, Neck, and Head [22,23]. The Backbone is used for extracting information from the input feature maps. The Neck fuses feature maps of different scales, while the Head classifies and localizes surface defects. These components work together to ensure effective detection capabilities. However, YOLOv10n demonstrates limitations in PCB defect detection tasks. The standard convolutions and fixed structures in its backbone network have insufficient feature extraction capability for small and irregularly shaped defects, resulting in missed detections and localization deviations. Additionally, its loss function exhibits limited optimization efficiency for complex samples, further constraining the overall performance improvement. Thus, enhancements to the baseline model are required.

## 4. Improved EDF-YOLOv10 algorithm

**Fig 4:**
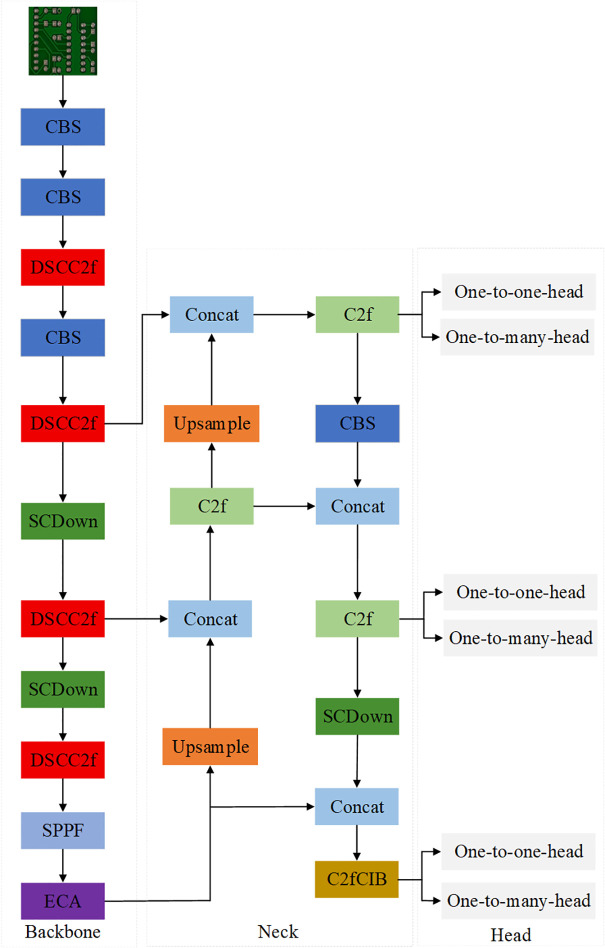
Structure of EDF-YOLOv10n network.

To address challenges such as high miss detection rates and slow model convergence caused by indistinct small defect features and complex background interference in PCB defect detection, we propose an improved EDF-YOLOv10n algorithm. The architecture of our proposed model is illustrated in Fig 4. This study focuses on enhancing the YOLOv10 backbone network by incorporating an ECA mechanism and DSCC2f module. This improvement aims to enhance the model’s feature extraction capabilities, particularly for slender and curved small-target defects. Additionally, we introduce the Focaler-CIoU loss function, which dynamically adjusts sample weights to optimize detection performance for hard samples effectively. The improved network demonstrates greater accuracy and robustness in detecting defects on PCBs.

### 4.1. Efficient channel attention mechanism

In PCB defect detection tasks, the feature signals of small defects are easily masked by complex background textures. This significantly hinders the learning process of the model. The original PSA module in YOLOv10 utilizes feature reorganization and a multi-branch design to integrate spatial and channel information, resulting in high computational complexity. Furthermore, its spatial attention branch further erodes the detailed information of small defects in deep feature maps. This increases the risk of missing small defects and limits the model’s adaptability for detecting small defects on PCBs. Therefore, the PSA module is replaced with the ECA module [24]. This module compresses spatial information through global average pooling and achieves lightweight cross-channel interaction by utilizing one-dimensional convolution with an adaptive kernel size. It enhances the semantic response of small defects in deep feature maps while preserving the complete channel structure, making it suitable for PCB defect detection tasks.

**Fig 5:**
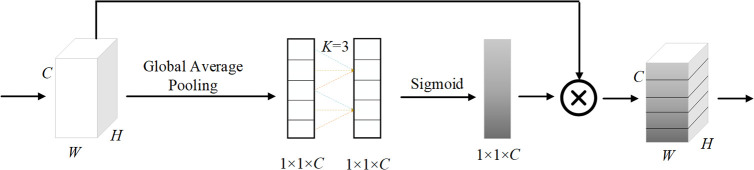
Structure of ECA module.

ECA is an efficient channel attention mechanism and it models inter-channel dependencies through a lightweight one-dimensional convolution applied dynamically. ECA first performs global average pooling on the input feature map and then uses a one-dimensional convolution with an adaptive kernel size to achieve cross-channel interaction. After that, it generates channel attention weights using the Sigmoid activation function and then multiplies the input feature map by these weights to obtain the output. The structure of ECA is shown in Fig 5, where *W*, *H*, and *C* represent the width, height, and number of channels of the feature map, respectively. *K* is the adaptive kernel size, and⊗ denotes element-wise multiplication.

### 4.2. DSCC2f module

**Fig 6:**
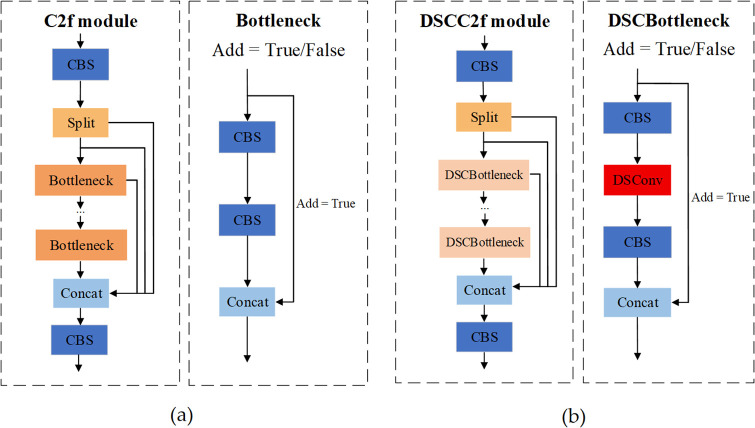
Structure of C2f and DSCC2f module. **(a)** structure of C2f module; **(b)** structure of DSCC2f module.

The C2f module of YOLOv10 is a key component for feature extraction in the backbone network. It divides the feature map into two parallel branches: one branch focuses on extracting deep semantic features using multiple Bottleneck blocks, while the other branch preserves shallow details through a short path. These two sets of features are then concatenated together. The structure is illustrated in Fig 6(a). PCB defects exhibit complex and variable geometric structures. The standard convolution in C2f employs a fixed rectangular sampling grid. This grid’s fixed receptive field struggles to capture such complex structures, and its static sampling points cannot dynamically adjust to the target shape. Consequently, shape features are lost, and the deviation of the feature extraction region from the main defect area ultimately leads to localization errors. To address this, this study introduces DSConv into the C2f module, constructing the DSCC2f structure shown in Fig 6(b).

**Fig 7:**
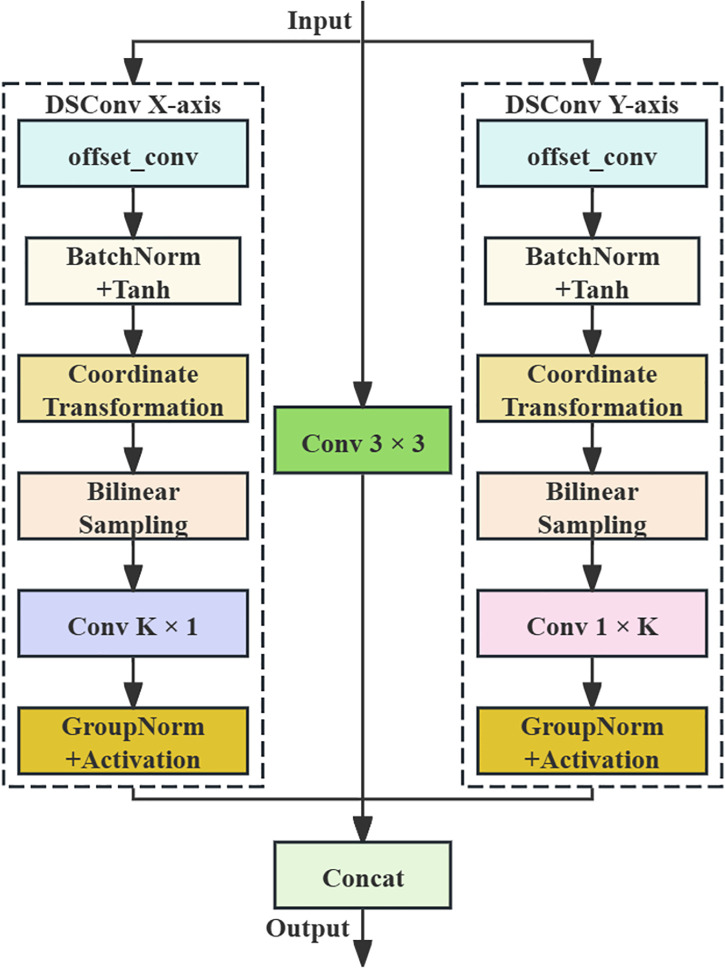
Structure of DSConv module.


DSConv adapts to tubular structures through iterative accumulation of offsets, allowing for better focus on slender and curved local structural features [25]. The workflow of DSConv is shown in Fig 7. This module uses standard convolution to extract basic features, employs dynamic snake convolutions along the X and Y axes to extract features, and concatenates the three branches in the channel dimension to achieve multi-view feature fusion. The principle of feature extraction in the X direction is as follows: The input feature map generates offset values through offset convolution, and the offset values are processed using BatchNorm and the hyperbolic tangent function. These offsets are then used to perform coordinate transformation on the convolution kernel’s sampling grid. Bilinear Sampling is applied to determine the sampling positions of the deformed convolution kernel on the input feature map. Finally, the K × 1 convolution operation is performed on the deformed feature map to extract local features, and groupnorm and an activation function are used to enhance feature expression capabilities. Assuming a standard 2D convolution (kernel size = 3) as Z, the central grid coordinate is $Z_{i}=\left(x_{i},y_{i}\right)$. Stretch Z along the X direction, $Z_{i\pm c}=\left(x_{i\pm c},y_{i\pm c}\right)$ represents the coordinates of $Z_{i}$ after shifting left or right by *c* units, its formula is

$$Z_{i\pm c}=\left\{\begin{array}{c} @l@\left(x_{i}+c,y_{i}+\sum_{i}^{i+c}\Delta y\right)\\ \left(x_{i}-c,y_{i}+\sum_{i-c}^{i}\Delta y\right) \end{array}\right\},$$ (1)

where *c* denotes the horizontal distance from this grid to the central grid, c={0,1,2,3,4}; *∆y* represents the offset in the *y*-axis direction. DSConv enhances the model’s ability to capture PCB defect features by dynamically predicting and fitting the serpentine sampling path of the target geometric structure while adopting a multi-scale feature fusion strategy.

### 4.3. Optimizing loss function of the EDF-YOLOv10 model

The design of the loss function is one of the important factors affecting model performance. YOLOv10n employs the CIoU loss function, and the calculation expression is [26]


$$L_{CIoU}=1-IoU+\frac{\rho^{\text{2}}\left(b_{pred},b_{gt}\right)}{c^{\text{2}}}+\alpha v,$$ (2)

where IoU represents the intersection over union of the predicted box and the ground-truth box; *bpred* and *bgt* denote the center coordinates of these boxes, $\rho \left(b_{pred},b_{gt}\right)$ represents the euclidean distance between the two centers; *c* is the diagonal length of the smallest enclosing rectangle that bounds both the predicted box and the ground-truth box; *v* is used to measure the similarity of the aspect ratios between these boxes; α is the weight coefficient.

In PCB defect datasets, small and irregularly shaped defects occupy a few pixels or have complex contours. Their predicted bounding boxes generally yield low IoU values with ground truth boxes during regression. Consequently, they are typically treated as complex samples during training. However, CIoU is insufficiently sensitive to the problem of sample distribution imbalance. When a dataset contains a large number of easy samples and a few hard samples, the model tends to focus on the easy samples while neglecting the hard samples, resulting in limited performance improvement. To better address the issue of sample distribution imbalance, the Focaler-IoU loss function [27] is introduced. It reconstructs the IoU loss using a linear interval mapping method, allowing the model to focus on samples of varying difficulty levels dynamically. The Focaler-IoU loss function is


$$L_{focaler-IoU}=1-IoU^{focaler},$$ (3)

$$IoU^{focaler}=\left\{ \begin{array}{c} @l@0,IoU<d\\ \frac{\left(IoU-d\right)}{u-d},d\ll IoU\ll u\\ 1,IoU>u \end{array}\right..$$ (4)

In Equation (4), the threshold parameters *d* and *u* (with values between 0 and 1) are used to adjust the sensitivity of the loss function. The Focaler-CIoU loss function is formed by combining the Focaler-IoU with the original CIoU loss function. This loss function enhances the accuracy and efficiency of PCB defect detection by incorporating dynamic sample weighting and geometric constraint mechanisms, and its formula is


$$L_{focaler-CIoU}=L_{CIoU}+IoU-IoU^{focaler}.$$ (5)

## 5. Experiments and result analysis

The configuration of the experimental environment is as follows: the central processing unit (CPU) is an Intel(R) Core(TM) i7-10700, the graphics processing unit (GPU) is an NVIDIA Quadro P2200, and the operating system is Windows 10. The deep learning framework is PyTorch 2.0.1, and the CUDA version is 11.8. The training parameters are set as follows: the input image size is 640 × 640 pixels, the number of training epochs for the YOLO model is 150, and the batch size is 4. The initial learning rate starts at 0.01 and decreases to a final rate of 0.0001. The Stochastic Gradient Descent (SGD) algorithm is used, with a momentum factor of 0.937. The validation period is set at every 10 epochs, and no pre-trained weights are employed during the training process.

### 5.1. Evaluation metrics

The experimental evaluation metrics include precision (P), recall (R), average precision (AP), mean average precision (mAP), number of parameters, and floating point operations per second (FLOPs). The calculation expressions for P, R, AP, and mAP are as follows:


$$\begin{array}{c} P=\frac{TP}{TP+FP},R=\frac{TP}{TP+FN},\\ AP=\int_{\text{0}}^{\text{1}}P\left(R\right)dR,mAP=\frac{\text{1}}{N}\sum_{i=1}^{N}AP_{i}. \end{array}$$ (6)

where *TP* and *FP* represent the number of defects detected accurately and incorrectly, respectively, and *FN* indicates the count of positive samples that are incorrectly predicted as negative by the model; *AP* denotes the area enclosed by the Precision-Recall curve and the coordinate axes; *N* represents the count of input feature maps, and *APi* denotes the average precision for each category with *i* as the category index; mAP@0.50 refers to the mean *AP* when the IoU threshold is 0.5 and mAP@0.50:0.95 denotes the mean *AP* across IoU thresholds from 0.50 to 0.95 with a step size of 0.05.

### 5.2. EDF-YOLOv10 model detection results and analysis

To validate the effectiveness of the improved EDF-YOLOv10 algorithm for PCB defect detection, this study compares the mAP@0.50, mAP@0.50:0.95, and the AP values for various defect types on the experimental dataset before and after the improvement. The comparison values of the single experiment results are shown in Table 1. It can be observed that the EDF-YOLOv10 algorithm improves the detection accuracy for open circuit, short, spur, and spurious copper defects by 8.9, 3.2, 4.5, and 6.6 percentage points, respectively, compared with the YOLOv10 model. In terms of mAP@0.50 and mAP@0.50:0.95, the improved model attains 90.6% and 48.4%. These values exceed those of the baseline model by 3.0 and 1.6 percentage points, respectively. These results demonstrate that the EDF-YOLOv10 model exhibits higher detection accuracy in PCB defect detection tasks, verifying the effectiveness of the improved algorithm.

**Table 1:** Comparison of detection accuracies between EDF-YOLOv10 and YOLOv10.

Model	mAP@0.50 (%)						mAP@0.50 (%)	mAP@0.50:0.95 (%)
	missing_hole	mouse_bite	open_circuit	short	spur	spurious_copper		
YOLOv10	99.5	94.6	77.3	92.2	72.7	89.5	87.6	46.8
EDF-YOLOv10	99.5	89.1	86.2	95.4	77.2	96.1	90.6	48.4

**Fig 8:**
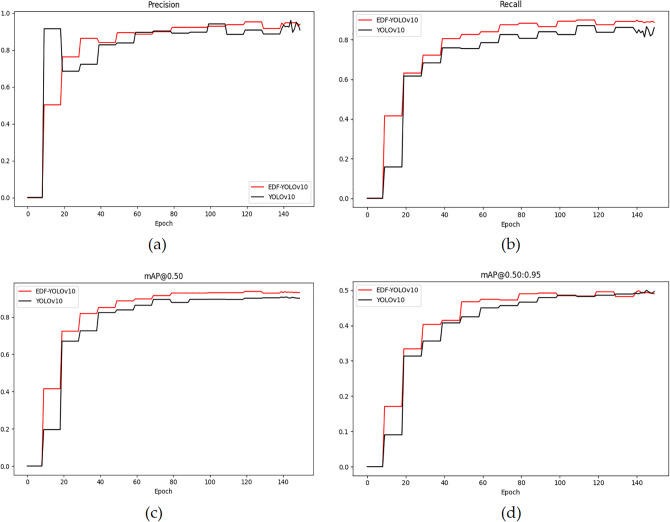
Comparison of EDF-YOLOv10 and YOLOv10 indices. **(a)** precision; **(b)** recall; **(c)** mAP@0.50; **(d)** mAP@0.50:0.95.

Fig 8 illustrates the variations of four metrics: precision, recall, mAP@0.50, and mAP@0.50:0.95 during the training process of EDF-YOLOv10 and YOLOv10. As shown in Figs 8(a) and 8(b), the precision and recall of EDF-YOLOv10 increase rapidly in the initial training stages and stabilize at higher values, with overall curves consistently above those of YOLOv10. The variations in mAP@0.50 and mAP@0.50:0.95, displayed in Figs 8(c) and 8(d), indicate that the values for EDF-YOLOv10 consistently surpass those of the YOLOv10 model, demonstrating a faster convergence speed and higher detection accuracy.

**Fig 9:**
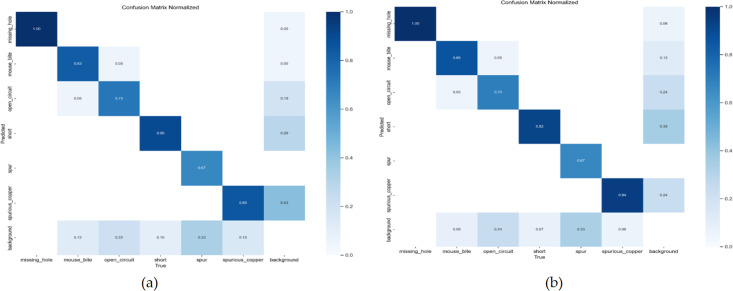
Comparison of confusion matrices between YOLOv10 and EDF-YOLOv10. **(a)** YOLOv10; **(b)** EDF-YOLOv10.

**Fig 10:**
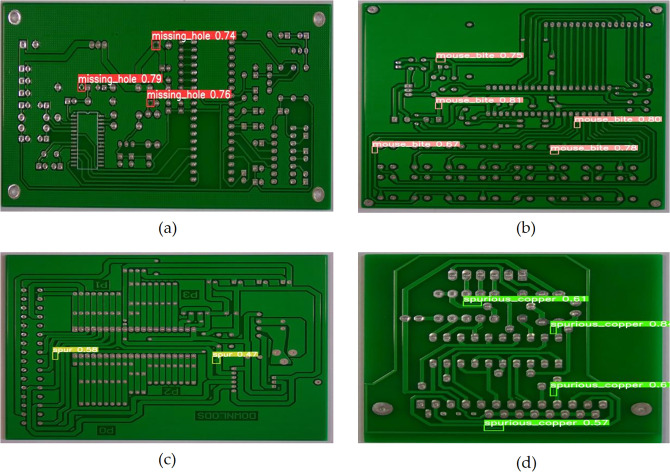
Detection results of YOLOv10. **(a)** missing hole; **(b)** mouse bite; **(c)** spur; **(d)** spurious copper.

**Fig 11:**
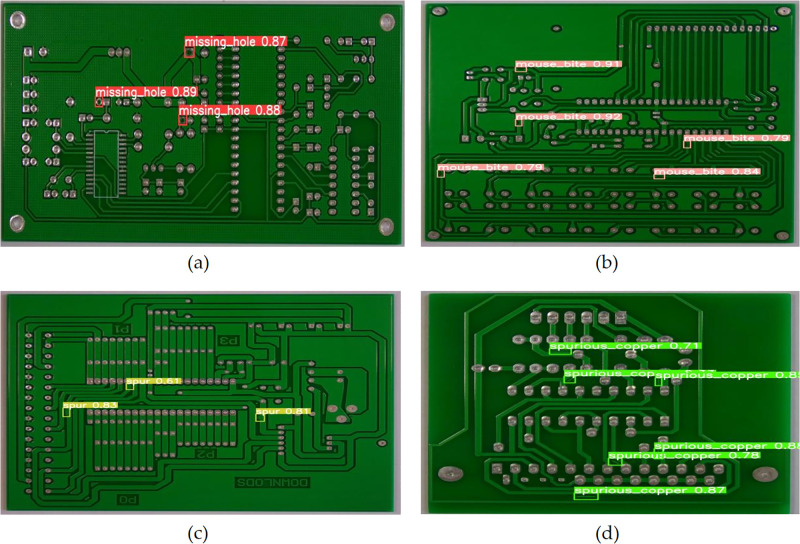
Detection results of EDF-YOLOv10. **(a)** missing hole; **(b)** mouse bite; **(c)** spur; **(d)** spurious copper.

The normalized confusion matrix demonstrates the relationship between the model’s predicted categories and the actual categories. The diagonal elements represent the ratio of correct predictions for each category. Fig 9 shows the normalized confusion matrices of YOLOv10 and EDF-YOLOv10. It can be seen that the diagonal values of the EDF-YOLOv10 model for mouse bite, short, and spurious copper defect categories are all higher than those for the YOLOv10 model. This indicates that the improved model exhibits better classification performance for these defect categories. Figs 10 and 11 present the visual detection results of YOLOv10 and EDF-YOLOv10 on the test dataset, respectively. As shown in Figs 10 and 11 (a) and (b), the confidence scores of EDF-YOLOv10 for missing hole and mouse bite defects are generally higher than those of YOLOv10, indicating that its detection results are more reliable. In Figs 10 and 11 (c) and (d), the number of spur and spurious copper defects detected by EDF-YOLOv10 exceeds that detected by YOLOv10, suggesting a lower missed detection rate. These results show that the improved model can more effectively identify and localize various defects in PCB defect detection tasks.

### 5.3. Statistical validation of experimental results

Five independent repeated training runs are conducted for both the YOLOv10 and EDF-YOLOv10 models to verify the reliability of the experimental results. The Bootstrap resampling method, suitable for small-sample analysis, is used to calculate the 95% confidence intervals for mAP@0.50 and mAP@0.50:0.95. This non-parametric method constructs an empirical distribution of the statistics through repeated resampling of the experimental data. The 2.5th and 97.5th percentiles establish the confidence limits, providing a robust foundation for statistical significance comparison in small-sample scenarios.

Table 2 displays the statistical results from the five repeated experiments. The EDF-YOLOv10 model showed 0.6% standard deviation for mAP@0.50 and mAP@0.50:0.95. This indicates minimal performance fluctuation and good training stability. The 95% confidence interval for mAP@0.50 ([89.6%, 90.6%]) does not overlap with that of YOLOv10 ([87.1%, 88.2%]); similarly, for mAP@0.50:0.95, the interval for EDF-YOLOv10 ([47.4%, 48.3%]) does not overlap with that for YOLOv10 ([45.7%, 46.6%]). The non-overlap in confidence intervals indicates a statistically significant performance improvement for EDF-YOLOv10, rather than one due to random variance. Furthermore, the mean mAP@0.50 and mAP@0.50:0.95 values for EDF-YOLOv10 across five runs were 90.1% and 47.9%, respectively. These represent gains of 2.4 and 1.7 percentage points over the baseline model, confirming the effectiveness of the proposed improvement strategy.

**Table 2:** Comparison of detection performance between YOLOv10 and EDF-YOLOv10 from five experiments.

Model	mAP@0.50 (%)		mAP@0.50:0.95 (%)	
	Mean±Std	95% Confidence Interval	Mean±Std	95% Confidence Interval
YOLOv10	87.7 ± 0.7	[87.1, 88.2]	46.2 ± 0.6	[45.7, 46.6]
EDF-YOLOv10	90.1 ± 0.6	[89.6, 90.6]	47.9 ± 0.6	[47.4, 48.3]

### 5.4. Ablation experiments of adding specific module

To verify the performance improvement of the ECA, DSCC2f, and Focaler-CIoU modules, ablation experiments are conducted on the PCB dataset, with the results presented in Table 3. A “√” indicates the application of the corresponding improvement method to YOLOv10. When the ECA module is introduced alone, the model’s mAP@0.50 metric increased to 88.3%, while the count of parameters and computational complexity decreased, indicating that this module can efficiently enhance feature extraction capabilities while achieving model lightweight optimization. Integrating only the DSConv module improved the mAP@0.50 and precision to 88.1% and 95.7%, respectively, but the model’s parameter count and computational complexity increased due to its convolutional calculation mechanism. The addition of the Focaler-CIoU module alone improved the mAP@0.50 metric to 88.9% by optimizing the bounding box regression process. The integration of any two modules further improves the mAP@0.50 metric, demonstrating effective synergy among the different components. When all three modules are integrated, the model achieves the highest values in both mAP@0.50 and mAP@0.50:0.95 metrics, reaching 90.6% and 48.4%, respectively, with a parameter count of 2.9M and a computational complexity of 9.0 GFLOPs. This demonstrates that the combination of the three modules improves PCB detection accuracy while maintaining a low computational overhead.

**Table 3:** Results of the ablation experiment.

ECA	DSCC2f	Focaler-CIoU	mAP@0.50 (%)	mAP@0.50:0.95 (%)	Precision (%)	Recall (%)	Parameters(M)	FLOPs(G)
			87.6	46.8	93.9	80.8	2.7	8.2
√			88.3	47.9	87.9	86.5	2.4	8.0
	√		88.1	47.8	95.7	79.7	3.1	9.2
		√	88.9	47.1	93.6	84.5	2.7	8.2
√	√		90.4	47.6	94.5	84.3	2.9	9.0
√		√	90.1	47.3	93.7	87.9	2.4	8.0
	√	√	90.1	46.8	93.6	85.3	3.1	9.2
√	√	√	90.6	48.4	94.2	82.9	2.9	9.0

### 5.5. Comparative analysis of loss functions

To validate the effectiveness of loss function improvements in enhancing PCB defect detection performance, this study compares the performance of various loss functions that integrate ECA and DSConv modules. As shown in Table 4, Focaler-CIoU achieves the highest values in both mAP@0.50 and mAP@0.50:0.95 metrics, reaching 90.6% and 48.4%, respectively. This demonstrates that the improved loss function enhances the model’s ability to detect PCB defects. Focaler-SIoU achieves 47.6% in the mAP@0.50:0.95, surpassing the SIoU loss function by two percentage points. Moreover, Focaler-ShapeIoU reaches an accuracy of 90.0% in mAP@0.50, outperforming ShapeIoU. These results further demonstrate that the Focaler-IoU mechanism can enhance the model’s detection accuracy.

**Table 4:** Performance comparison of different loss functions for YOLOv10 model with ECA and DSConv enhancements.

Loss Function	mAP@0.50 (%)	mAP@0.50:0.95 (%)	Precision (%)	Recall (%)
EIoU [28]	80.1	41.0	82.2	72.5
SIoU [29]	88.5	45.6	92.3	86.4
ShapeIoU [30]	89.4	47.5	94.4	82.9
CIoU [26]	90.4	47.6	94.5	84.3
Focaler-IoU [27]	90.6	46.9	91.5	86.3
Focaler-SIoU	88.2	47.6	90.9	84.6
Focaler-ShapeIoU	90.0	47.5	90.4	85.3
Focaler-CIoU	90.6	48.4	94.2	82.9

### 5.6. Comparison experiment of different detection models

Comparative experiments are conducted on the augmented PKU-Market-PCB and DeepPCB datasets [31] against three categories of mainstream object detection models: (1) mainstream YOLO series models, including YOLOv3 [32], YOLOv6n [33], YOLOv7-tiny [34], YOLOv8n, YOLOv10n [21], and YOLOX-tiny [35]; (2) other single-stage models, including Rtmdnet_tiny and Rtmdnet_s; (3) the Transformer-based detection model Deformable-DETR [36].

The test results on the augmented PKU-Market-PCB dataset are shown in Table 5. Compared to the mainstream YOLO series, EDF-YOLOv10n surpasses YOLOv3, YOLOv6n, and YOLOv7-tiny in mAP@0.50 by 14.7, 1.9, and 6.9 percentage points, respectively, while utilizing fewer parameters and lower computational cost. It also outperforms YOLOv8n, YOLOv10n, and YOLOX-tiny by 1.4, 3.0, and 3.9 percentage points in mAP@0.50, demonstrating a clear accuracy advantage. Among other single-stage models, EDF-YOLOv10n achieves gains of 2.4 and 1.7 percentage points in mAP@0.50 over Rtmdnet_tiny and Rtmdnet_s, respectively. Compared to the Transformer-based model Deformable-DETR, the proposed method improves mAP@0.50 by 0.8 percentage points, while reducing parameters and computation by 37.1M and 164 FLOPs. The experiments indicate that EDF-YOLOv10n achieves the best detection accuracy among all compared models, showcasing outstanding overall performance.

**Table 5:** Comparative experimental results for different models on the PKU-Market-PCB dataset.

Model	mAP@0.50 (%)	Precision (%)	Recall (%)	Parameters (M)	FLOPs (G)
YOLOv3	75.9	84.7	75.4	61.6	15.9
YOLOv6n	88.7	91.7	84.9	4.6	11.3
YOLOv7-tiny	83.7	94.7	75.1	6.0	13.1
YOLOv8n	89.2	93.1	83.5	3.0	8.1
YOLOv10n	87.6	93.9	80.8	2.7	8.2
YOLOX-tiny	86.7	95.6	78.0	5.1	6.5
Rtmdet_tiny	88.2	97.3	81.8	4.8	8.1
Rtmdet_s	88.9	95.9	81.5	8.9	14.8
Deformable-DETR	89.8	86.7	86.1	40.0	173
EDF-YOLOv10n	90.6	94.2	82.9	2.9	9.0

The test results on the DeepPCB dataset are presented in Table 6. Compared to mainstream YOLO series models, EDF-YOLOv10n outperforms YOLOv3, YOLOv6n, and YOLOv7-tiny in mAP@0.50 by 0.7, 3.6, and 7.6 percentage points, respectively. It also surpasses YOLOv8n, YOLOv10n, and YOLOX-tiny in mAP@0.50 by 1.2, 0.8, and 0.4 percentage points, with both precision and recall rates exceeding those of these models. Among the Rtmdnet series models, EDF-YOLOv10n matches Rtmdnet_tiny in mAP@0.50 but achieves higher precision and recall by 0.2 and 0.3 percentage points, respectively, while reducing the parameter count by 1.9M. Compared to Rtmdnet_s, it improves mAP@0.50 by 0.2 percentage points. For Transformer-based detectors, the proposed model increases mAP@0.50 by 0.9 percentage points over Deformable-DETR. The experimental results demonstrate that EDF-YOLOv10n achieves superior detection accuracy on both the PKU-Market-PCB and DeepPCB datasets, exhibiting excellent generalization capability and overall performance.

**Table 6:** Comparative experimental results for different models on the DeepPCB dataset.

Model	mAP@0.50 (%)	Precision (%)	Recall (%)	Parameters (M)	FLOPs (G)
YOLOv3	97.9	95.9	96.1	61.6	15.9
YOLOv6n	95.0	93.4	88.5	4.6	11.3
YOLOv7-tiny	91.0	85.3	86.2	6.0	13.1
YOLOv8n	97.4	94.5	93.7	3.0	8.1
YOLOv10n	97.8	96.1	94.0	2.7	8.2
YOLOX-tiny	98.2	96.5	95.5	5.1	6.5
Rtmdet_tiny	98.6	96.6	95.3	4.8	8.1
Rtmdet_s	98.4	96.6	95.9	8.9	14.8
Deformable-DETR	97.7	96.0	91.9	40.0	173
EDF-YOLOv10n	98.6	96.8	95.6	2.9	9.0

### 5.7. Simulation experiment based on EDF-YOLOv10

**Fig 12:**
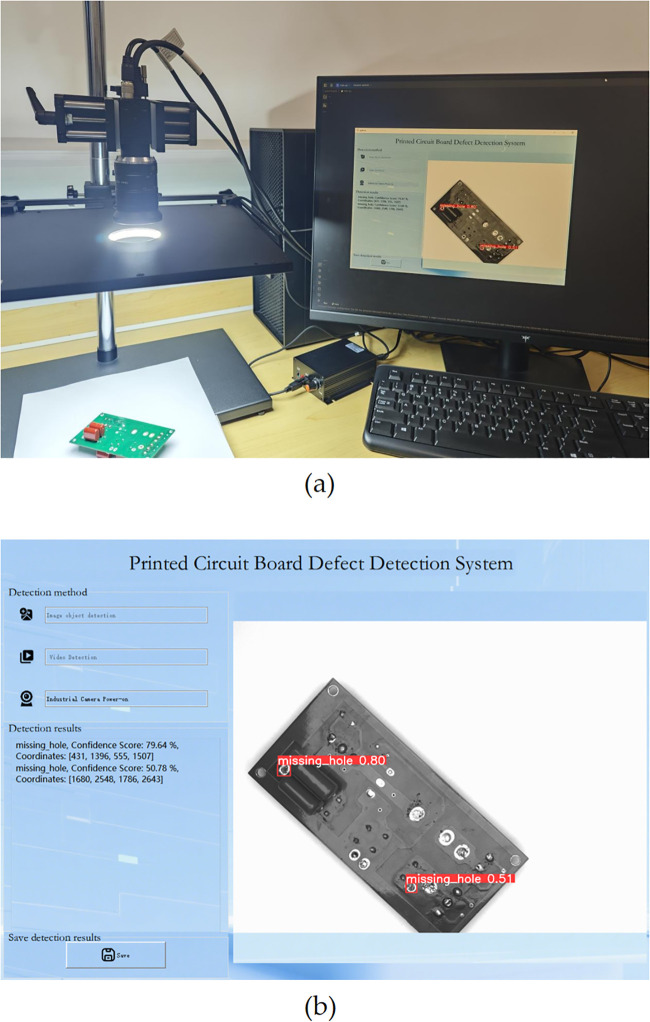
PCB simulation experimental results. **(a)** hardware system; **(b)** detection results on the visualization interface.

The hardware system incorporates industrial cameras, surface lighting, and light controllers combined with a PyQt5-based visualization interface to enable PCB surface defect detection. Fig 12 presents the detection results for the missing hole, demonstrating that the system effectively executes the defect detection task using the EDF-YOLOv10 algorithm.

## 6. Conclusions

To address the issues of low detection accuracy for small targets and hard samples on PCB surfaces, as well as slow model convergence in the YOLOv10 algorithm, this paper proposes the EDF-YOLOv10 algorithm. The ECA mechanism enhances the weak channel feature responses of small PCB defects and suppresses background noise interference, addressing the insufficient feature extraction for small defects in existing YOLOv10 improvements. The improved DSCC2f module dynamically adjusts the sampling positions of convolution kernels to flexibly adapt to the geometric contours of irregular PCB defects, compensating for the inadequate feature representation of irregular defects by traditional rectangular convolutions. The Focaler-CIoU loss function is optimized for the imbalanced sample distribution in PCB defects. It enhances the discriminative ability for low IoU samples and accelerates model convergence, overcoming the slow convergence and insufficient learning of complex samples in current YOLOv10 enhancements. Through the collaborative design of the ECA attention mechanism, the DSConv module, and the Focaler-CIoU loss function, the improved YOLOv10 framework proposed in this work simultaneously enhances small defect detection, irregular defect adaptation, and model convergence speed. This integrated approach effectively solves the multi-dimensional challenges that existing methods struggle to address. Experimental results show that the improved algorithm achieves mAP@0.50 and mAP@0.50:0.95 of 90.6% and 48.4%, respectively, representing improvements of 3.0 and 1.6 percentage points compared to the original YOLOv10. With a parameter count of 2.9M and a computational complexity of 9.0 GFLOPs, the model achieves a balance between high accuracy and a lightweight design. Additionally, the defect detection system constructed in this study leverages the improved algorithm to detect PCB surfaces, demonstrating practical application value. Future research will explore more lightweight detection algorithms and apply the improved algorithm to PCB defect detection on embedded platforms.
